# A Secure Scheme Based on a Hybrid of Classical-Quantum Communications Protocols for Managing Classical Blockchains

**DOI:** 10.3390/e25050811

**Published:** 2023-05-17

**Authors:** Ang Liu, Xiu-Bo Chen, Shengwei Xu, Zhuo Wang, Zhengyang Li, Liwei Xu, Yanshuo Zhang, Ying Chen

**Affiliations:** 1 Information Security Center, State Key Laboratory of Networking and Switching Technology, Beijing University of Posts and Telecommunications, Beijing 100876, China; liuang0826@163.com; 2 Beijing Electronic Science and Technology Institute, Beijing 100070, China; 3 School of Artificial Intelligence, Beijing University of Posts and Telecommunications, Beijing 100876, China

**Keywords:** quantum blockchain, quantum signature, QPoA, post-quantum

## Abstract

Blockchain technology affords data integrity protection and building trust mechanisms in transactions for distributed networks, and, therefore, is seen as a promising revolutionary information technology. At the same time, the ongoing breakthrough in quantum computation technology contributes toward large-scale quantum computers, which might attack classic cryptography, seriously threatening the classic cryptography security currently employed in the blockchain. As a better alternative, a quantum blockchain has high expectations of being immune to quantum computing attacks perpetrated by quantum adversaries. Although several works have been presented, the problems of impracticality and inefficiency in quantum blockchain systems remain prominent and need to be addressed. First, this paper develops a quantum-secure blockchain (QSB) scheme by introducing a consensus mechanism—quantum proof of authority (QPoA) and an identity-based quantum signature (IQS)—wherein QPoA is used for new block generation and IQS is used for transaction signing and verification. Second, QPoA is developed by adopting a quantum voting protocol to achieve secure and efficient decentralization for the blockchain system, and a quantum random number generator (QRNG) is deployed for randomized leader node election to protect the blockchain system from centralized attacks like distributed denial of service (DDoS). Compared to previous work, our scheme is more practical and efficient without sacrificing security, greatly contributing to better addressing the challenges in the quantum era. Extensive security analysis demonstrates that our scheme provides better protection against quantum computing attacks than classic blockchains. Overall, our scheme presents a feasible solution for blockchain systems against quantum computing attacks through a quantum strategy, contributing toward quantum-secured blockchain in the quantum era.

## 1. Introduction

Since the introduction of Bitcoin in 2008 [1], its underlying technology, the blockchain, has attracted significant research attention and has been widely applied in different fields, such as finance and medical health. In a typical blockchain system, a consensus mechanism elects a representative node for bookkeeping and to reach consistency on a new block; the signing and verification of a transaction are accomplished through a digital signature, and the integrity protection of the transaction records is achieved by a hash function. The accelerating development of quantum computing technology seriously threatens the security of classical blockchains. Specifically, this is reflected in three areas: attacks against consensus mechanisms, attacks against digital signatures, and attacks against hash functions. (1) Regarding consensus mechanisms, proof of work (PoW) is vulnerable to mining attacks [2], while proof of stake (PoS) exposes stakers to the risk of losing their assets in staking transactions [3], and the classic voting process in delegated proof of stake (DPoS) is susceptible to forging attacks [4]. (2) With regards to the security of transactions, classic blockchain systems often use public-key digital signatures whose security relies on complex computational problems that are hard for a classic computer to solve. Discrete logarithm factoring problems and large number decomposition problems are generally used to construct public-key signature algorithms, such as elliptic curve cryptography (ECC), Rivest–Shamir–Adleman (RSA), digital signature algorithm (DSA), and elliptic curve digital signature algorithm (ECDSA). However, these algorithms are vulnerable to quantum computing attacks, as the quantum algorithm—Shor’s algorithm [5]—can solve discrete logarithms or large-number decomposition problems in polynomial time. Thus, an attacker using Shor’s algorithm can retrieve the private key from the signature and the public key. Therefore, these public-key digital signatures will be insecure when the adversary has access to a quantum computer in the quantum era. (3) Regarding the data security of transaction records, classical hash functions are vulnerable to pre-image collision attacks, typically, Grover’s algorithm [6] can be exploited to accelerate the search for collisions, perform tampering attacks and compromise the data integrity of blocks.

In order to eliminate the threat of quantum computing attacks on blockchain systems, scholars have conducted in-depth research. Several post-quantum signature schemes (PQS) have been proposed to address the threats brought by quantum adversaries, such as lattice-based, code-based, hash-based, multivariate-based, and hybrid schemes [7]. These PQS involve hard problems for the quantum adversaries to solve, and their security relies on computational complexity or other difficulties such as encoding. However, complex computation imposes a high computing resource overhead and low transaction throughput, reducing the efficiency of blockchain systems. Some post-quantum algorithms may be currently secure against quantum adversaries but this still cannot be strictly proved theoretically, and with the proposal of new stronger quantum algorithms, these PQS algorithms may be breached or threatened.

Unlike PQS algorithms, quantum signatures (QS) security is not based on mathematical complexity problems. Instead, the security of QS relies on the physical properties of quantum mechanics, e.g., the quantum no-cloning theorem [8] and the Heisenberg uncertainty principle [9], which is computing-independent.

Recent research proposes several quantum blockchain schemes to improve blockchain system security against quantum computing attacks. For instance, E. O. Kiktenko et al. [10] proposed a quantum-secured blockchain scheme based on a hybrid network that comprises a quantum key distribution (QKD) [11] network and a classic network. In their work, the original Byzantine fault tolerance (BFT) consensus mechanism is utilized to reach a consensus, and QKD is used for identity authentication. However, the drawback of BFT has limited their scheme’s scalability, as BFT efficiency declines sharply as the number of users increases. In 2019, D. Rajan and M. Visser [12] conceptually developed a quantum blockchain based on entanglement in time, which is quite theoretical and cannot be accomplished based on existing technology. In 2020, Gao et al. [13] introduced a quantum blockchain scheme based on DPoS and quantum entanglement, which assumed that each user has a quantum computer and thus currently has no practical value. Moreover, in 2021, Wen et al. [14] proposed a quantum blockchain scheme using a quantum hash function (QHF), a quantum swap test circuit, and quantum teleportation. However, the quantum swap test circuit fails to distinguish two different quantum strings with a non-negligible probability. Therefore the method’s data consistency checking is doubtful. In addition, in 2022, El-Latif et al. [15] proposed a blockchain framework that involved QHF based on a quantum walk model, where the QHF was used for block linking. Although the blockchain scheme achieved integrity and confidentiality for data in Internet of Things (IoT) devices, the consensus mechanism was not mentioned in the paper. In the same year, Li et al. [4] proposed a quantum blockchain scheme based on QDPoS, where all classical information of a block is encoded into a single qubit, and all the qubits (blocks) are entangled to form a chain. Given the practical difficulties of long-term storage of quantum entangled particles under current conditions, the verification of data integrity on this quantum blockchain is quite complex, resulting in their scheme still being somewhat impractical under current technological conditions. Moreover, the QS in their scheme is one-time, a signature generated by the signer can only be verified once and then disappears. If *n* verifiers want to verify the transaction, the signer needs to perform the signing process *n* times. The security of QS depends on the number of quantum public keys, resulting in inefficiency and high quantum resource overhead. In 2022, Ye et al. [16] proposed a quantum blockchain scheme—QboT—for IoT by a QS, in QBoT, quantum state public keys are stored in blocks which is considered to be unpractical using current technology, as “*Long-term data storage is not possible because decoherence in the quantum world rapidly corrupts information, making it very difficult to rely on quantum memories/storage*” [17]. In 2022, Wang et al. [18] proposed a blockchain scheme based on an improved DPoS consensus algorithm and a quantum signature. In their consensus, the voting process is somewhat neglected, their work would have been better if the post-quantum security of the voting protocol had been taken into consideration.

The above work has made useful explorations into post-quantum security of blockchain systems, yet the following problems still exist: (1) lack of a secure and efficient consensus mechanism, e.g., the BFT of [10] limits the scale of the blockchain system, the schemes of [16] and [18] are based on DPoS, which may be fragile to quantum computing attacks during the voting process, while the scheme of [15] lacks a consensus mechanism. (2) The schemes are not practical and efficient enough, such as the block structure design [4,16] that stores quantum state information will face the problem of suffering from noise interference, and the quantum swap test circuit has the problem of non-negligible error rate when judging two different quantum strings [14]. (3) The transaction verification process consumes large quantum resources or the signature scheme is not practical enough. For example, in the scheme of [4], a quantum state public key needs to be consumed for each verification of the signature of a transaction; in the scheme of [18], the signature can only be verified once by the designated verifier and cannot be reverified.

At the same time, the rapid development of quantum cryptography provides us with a key to unlock the toolbox for eliminating quantum computing attacks. Some quantum hash function schemes with good collision resistance have been proposed [19,20], and several secure quantum digital signature schemes based on the physical properties of quantum mechanics have been constructed [21,22,23]. All these quantum cryptography components open a new path for us to design a post-quantum secure blockchain scheme.

Inspired by the above work, this study proposes a quantum-secure blockchain scheme (QSB) by introducing an improved proof of authority (PoA) [24] consensus algorithm and an IQS. As the quantum state particles collapse after measurement, a signature in quantum states will always “disappear” after verification, leading to a new problem for the QS. Specifically, a signature generated by QS can be verified only once, disappear, and cannot be verified again after the one-time verification. Therefore, many QS schemes do not support verifying a signature multiple times, unlike a classic public-key signature that can be verified by different parties as often as required. In a QS scheme, if a third party other than the signer and the verifier wants to check the validity of a signature after verification, he will fail in obtaining the quantum-state signature because it disappeared in the first verification. This QS characteristic will restrain applying QS in the blockchain.

To address this issue, a QS with signature proof retained is needed. Therefore, this paper proposes an IQS scheme. Given the robustness of a QS to quantum computing attacks, it can be used for transaction signing in the blockchain, as although the IQS can be verified only once and will collapse after verification, the signature proof will be left, convincing an unrelated party that the signature has been verified successfully. A third party other than the designated verifier can check the validity of the signature by the proof. Thus, the trust in QSB is improved in that IQS overcomes the weakness of a general QS.

The main contributions of this paper are concluded as follows.

1. A quantum-secure blockchain scheme (QSB) is proposed by utilizing a consensus mechanism, QPoA, and IQS. The QPoA is leveraged for block generation while the IQS is deployed in transaction verification. The QPoA adopts a behavior-scoring mechanism to improve the reliability of the validating nodes, and the IQS utilizes the validating node as a trusted third party to verify a transaction. By combining QPoA and IQS, QSB achieves a perfect balance of post-quantum security and practicality.
2. A consensus algorithm—QPoA—is developed in QSB for new block generation. First, QPoA adopts the Knuth shuffling algorithm with QRNG to rearrange validating nodes in an unpredictable random manner, which offers non-transparency of the leader node’s identity and immunity from centralized attacks. Second, by deploying a quantum voting protocol instead of classical voting, the post-quantum security of new block generation is enhanced. Third, by adopting an updating mechanism of validating nodes in QPoA, the trustworthiness of validating nodes is improved, therefore the trust establishment between two users in a transaction is better achieved.
3. An IQS scheme with an arbitrator is utilized in QSB to improve the security and efficiency of transactions. By storing the verification proof of the transaction in IQS, re-verification for a transaction is achieved, solving the disputation of a lost quantum signature and making our IQS more trustworthy and practical.
4. Based on QSB, a digital document exchange platform is designed to secure digital documents’ transmission and record receiving and sending operations accurately. Security analysis demonstrates that QSB is secure from both classical and post-quantum perspectives. Overall, the proposed QSB is a secure, practical, and efficient blockchain solution in the post-quantum era.

The remainder of this paper is organized as follows. Section 2 constructs a quantum-secure blockchain (QSB) scheme based on the QPoA consensus mechanism and the IQS, QPoA adopts a quantum voting protocol and QRNG for leader node election and leverages a dynamic updating mechanism to maintain the authority of nodes. Section 3 represents a transaction process in QSB by IQS. Section 4 conducts a security analysis of the proposed blockchain scheme, and Section 5 discusses the usage of QSB in a digital document exchange application scenario. Finally, Section 6 concludes this work.

## 2. Quantum-Secure Blockchain Scheme

QKD is a protocol whereby two communicating parties establish a key by transmitting quantum state particles with the aim of sharing a string of keys (consisting of classical random bits, but also known as “quantum keys” because they are established quantumly) between the two parties. In 1984, Bennett and Brassard first introduced quantum cryptography and gave the first QKD protocol, the BB84 protocol [11]. After nearly forty years of development, various QKD protocols [25,26,27,28,29,30] have been proposed based on different quantum mechanical properties, and the security of some typical QKD protocols has been rigorously proven to be information-theoretic secure. Although some security threats against QKD such as eavesdropping [31] may exist due to the imperfect nature of physical devices, adjusting parameters to increase the proportion of decoy-state particles during the post-processing of QKD can effectively mitigate such threats. As both QKD and one-time pad (OTP) encryption algorithms are information-theoretic secure, they can be used in combination to achieve perfectly secure and confidential communications.

Practical research of QKD is also progressing rapidly. In 2021, Pan et al. demonstrated an air-to-ground quantum network. Based on the quantum satellite Micius, all users in the QKD network can communicate with each other over a distance of up to 4600 km through an integrated optical fiber and free-space QKD network [32]. In 2022, Li et al. demonstrated an experiment for quantum state transfer (QST) across a distance of over 1200 km based on the entanglement between two far-away parties utilizing the quantum satellite Micius [33].

QRNG technology [34] is developing rapidly to be applied to QKD. Currently, QRNG is in the process of commercialization and the QRNG chip is maturing. In 2022, Quantum eMotion, a Canadian company, announced the design completion of its first blockchain application of QRNG technology.

In the network architecture of QSB, there are two layers of network in the distributed system, a classical network and a QKD network. All nodes are connected to each other by an authenticated quantum channel, and they can also communicate with each other in a classical channel. Any two nodes can implement the QKD protocol to share secret keys.

### 2.1. Blockchain Structure of QSB

**Figure 1:**
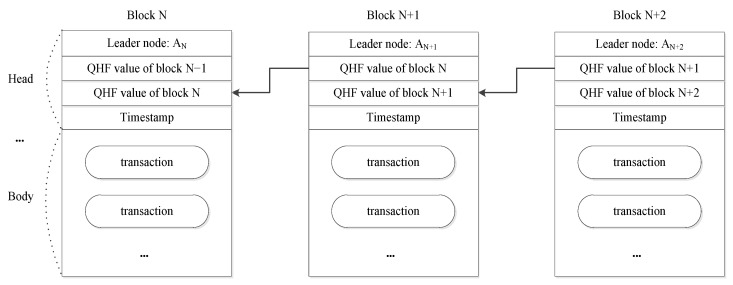
Structure of QSB.

The architecture of QSB is shown in Figure 1. Each block consists of two parts: a block head and a block body. The block head contains the block proposer who generated and proposed the block, the QHF value of the previous block, the timestamp of block generation, and other necessary information. The block body contains transactions arranged in chronological order by their timestamps. Each block stores the QHF value of its previous block, and all blocks are linked to form a chain structure in this way.

### 2.2. Consensus Mechanism in QSB

PoW and PoS are the mainstream consensus mechanisms of blockchain systems. The PoW consensus mechanism elects a bookkeeping node by solving a cryptographic puzzle (mining), i.e., the miner node that successfully solves the specified cryptographic puzzle first will receive the mining rewards and obtain bookkeeping rights, which is resource-consuming. In the PoS consensus mechanism, verifiers compete for bookkeeping rights of the new block by their stakes, and the one who has the most stake (including stake amount and holding time) will win the game and propose the new block. While PoS has good energy efficiency, there is a risk that the stake may become concentrated in the hands of a few participants, leading to a monopoly. The PoA is a blockchain consensus mechanism other than PoW and PoS, which Gavin Wood, the founder of Ethereum, first proposed. PoA is a reputation-based consensus mechanism that provides faster transactions. Based on the identity as a stake (IAS) mechanism, PoA provides a practical and efficient solution for blockchain networks (especially private blockchains). In QSB, an improved PoA consensus mechanism—QPoA—is developed.

The basic idea of PoA is to elect a central authority—leader node—to unify every node’s state, and the PoA network usually uses distributed accounting, where each node has a ledger. The consensus algorithm ensures that the state of each node is consistent to prevent inconsistency in everyone’s ledgers. The validator in the PoA network is the authority elected by everyone, and after the leader node proposes and validates a new block, the validating nodes synchronize the data from the leader node. PoA is a feasible consensus mechanism to maintain the consistency of all nodes’ states and effectively prevents double-spending attacks.

**Figure 2:**
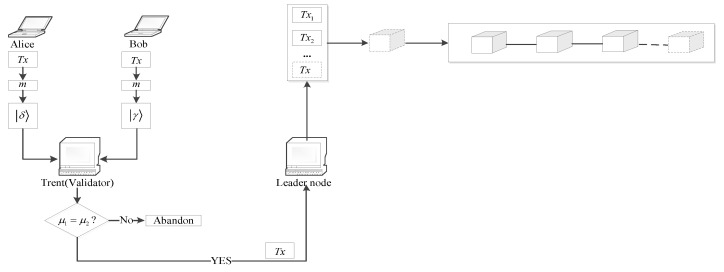
A transaction process.

In the blockchain system, when a leader node has proposed a new block and has packaged it into the blockchain, we define it as one consensus. When all nodes in the list of validating nodes (denoted as LV) have been elected as the leader node for one time, we define it as one consensus round. The transaction process in one consensus is shown in Figure 2.

Generating a new block in PoA is implemented by voting. All validating nodes vote on the candidate block proposed by the leader node, and the new block will be accepted and included in the blockchain if it gets at least $\left\lfloor \frac{V}{2}\right\rfloor +1$ affirmative votes (*V* is the number of validating nodes). Otherwise, it will be rejected.

Unlike mining in PoW, PoA maintains a list LV in the blockchain system, and the nodes in this list will generate a new block in turn. It should be noted that this is an energy-saving process. Regarding the PoA process, for instance, before a new consensus round begins, V nodes are elected as the validating nodes, and the LV is generated and kept in the blockchain system. According to the order of nodes in the LV, the role of the leader node is passed from one validating node to the next in turn. At the beginning of a consensus, one node in the LV will be elected as the leader node to generate a new block.

For better understanding, the mathematical symbols in the article are defined in the notation table, Table 1.

#### 2.2.1. The Election of Leader Node in a Consensus

In PoA, the order of nodes in the LV is kept constant, and the validating nodes take turns acting as the leader node by this fixed sequence. This will bring about the problem of over-exposure of the leader node’s identity, which will attract attacks. Once the leader node is attacked, the progress of new block generation will be delayed, thus reducing the efficiency of the whole blockchain system.

To tackle this issue, we introduce the Knuth shuffling algorithm [35] to elect the leader node randomly to conceal its identity in each consensus. By adopting the quantum multi-party secure computing method, all validating nodes participate together to share a public parameter *P_M_* before the start of the *M*th consensus round. In the *M*th consensus round, the newly generated leader node in each consensus is shared among all validating nodes by encryption of *P_M_*, which is only known to the validating nodes, and other unrelated parties including ordinary nodes know nothing about it.

Moreover, by utilizing the fundamental randomness of certain quantum processes [36], a QRNG can be used for random number generation in our scheme. The deployment of a QRNG in QPoA will make the leader node election totally unpredictable.

**Figure 3:**
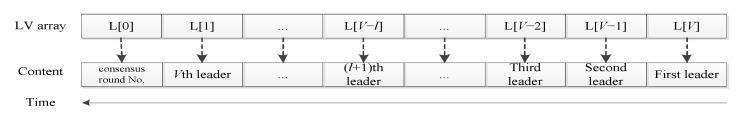
Content of LV array.

Specifically, to conceal the real identity of the leader node, we harness a QRNG [34] to reorder the LV, concealing the identity of the leader node in each consensus. In this way, the leader nodes can be effectively protected from concentrated attacks like DDoS due to the randomization of the leader node’s appearance order. As shown in Figure 3, each element of LV array stores the identity of a leader node.

The inputs are LV and *N* (*N* denotes the number of blocks in the current blockchain ledger), and the output is a reordered LV. t_N_ is the timestamp of block *N* that is used as the seed parameter for the QRNG. That is, t_N_ is used as a random seed of QRNG to generate a random number C[i] and the Knuth shuffling algorithm uses C[i] to generate a new leader node in the next consensus. The algorithm for the leader node selection is presented in Algorithm 1.
**Algorithm 1. The leader node selection algorithm**Input: *L*, *V*, *l*, *t_N_* //*L* is an array to store LV, each element stores an ID of a validating node; *V* denotes the number of elements in LV; *l* denotes the number of validating nodes that have already acted as the leader node in a consensus round; t*_N_* denotes the timestamp of the latest block B*_N_*.Output: *L*; *L*[i]int i,j;i = *V*-*l*;{  C[i] = QRNG(*ID*_(*V*-*l*)_,*t_N_*); // *ID*_(*V*-*l*)_ denotes identity of the current leader node  j = C[i]%(i+1);    tmp = *L*[i];    // *L*[i] stores ID of the i-th validating node in LV  *L*[i] = *L*[j];  *L*[j] = tmp;}if(l < V)  *l* = *l* + 1;else  *l* = 0;return *L*[i]; //The element in ith position in *L*, that is, *L*[i] stores the identity *ID_L_*_[i]_will be elected as the leader node in the forthcoming (*K*+1)th consensus

**Figure 4:**
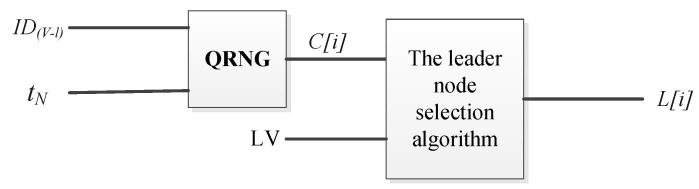
The process of the leader node election in one consensus.

The newly generated list LV determines the leader node in the forthcoming consensus. The exact process of the leader node election in one consensus is shown in Figure 4.

**Figure 5:**
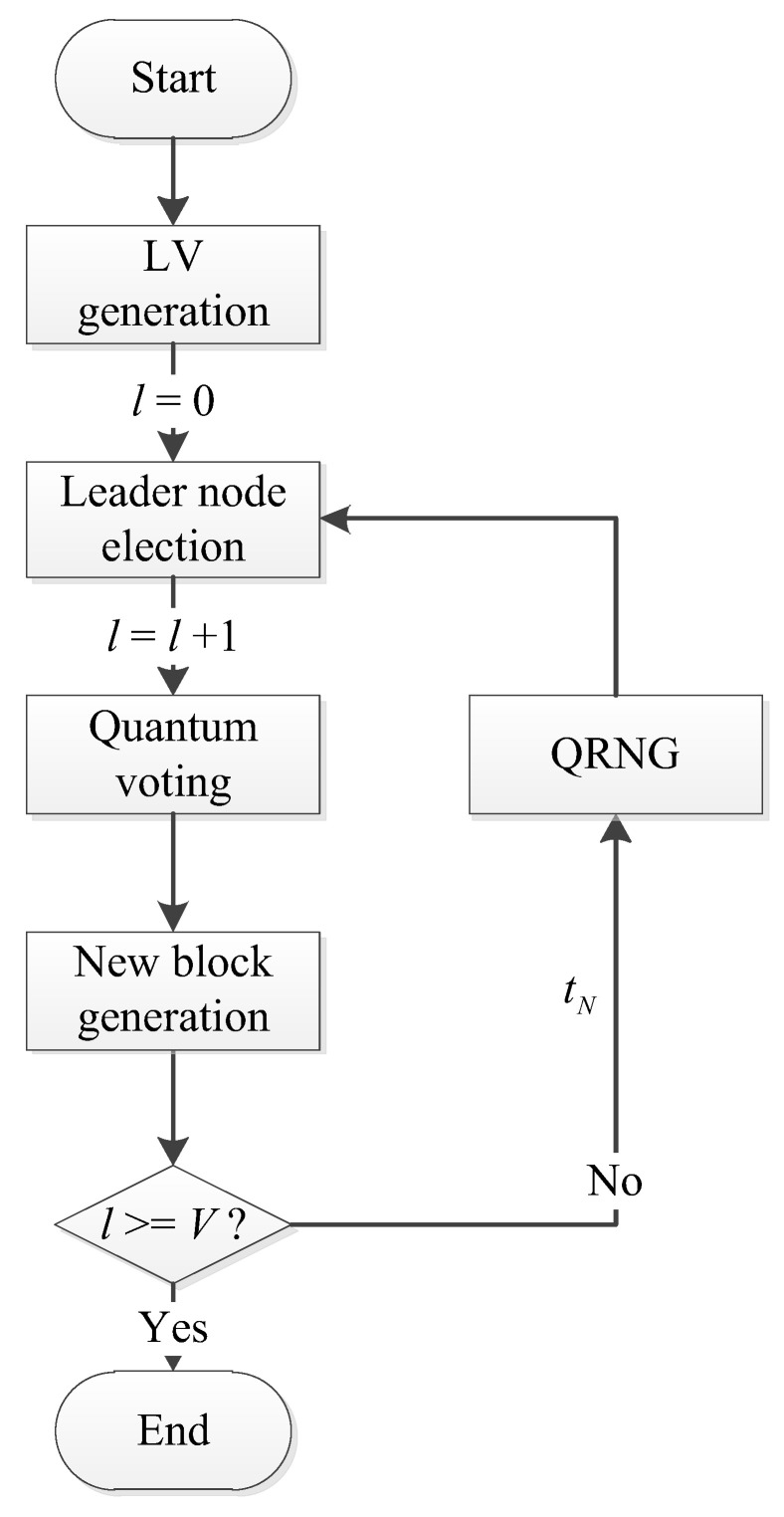
One consensus round in QPoA.

In each consensus round, all validating nodes use a verifiable multi-party QKD protocol [37] to secretly share a classical public key *P*, i.e., the shared parameter in the *M*th consensus round is *P_M_*. In a consensus of the *M*th consensus round, a leader node *ID_l_* is elected by the reordering algorithm in Algorithm 1, then *P_M_* is used to encrypt the identity of the new leader node *ID_l_* and *ID_l_* is published among all validating nodes in a secure quantum channel. Figure 5 illustrates the leader node election in a complete consensus round by QPoA.

#### 2.2.2. Generation of a New Block by Quantum Voting

In PoA, the leader node proposes a new block and sends it to all validating nodes for validation. The acceptance of a new block is implemented through classical voting. However, classical voting usually relies on a classic public-key cryptographic algorithm. For instance, in the PoA-based blockchain system Apla, the leader node signs a new block with a private key by the ECDSA algorithm in the voting process. This is quite insecure when quantum adversaries are considered due to the post-quantum vulnerability of classic public-key cryptosystems. In contrast, this problem does not exist in a quantum voting scheme since its security is guaranteed by the principles of quantum mechanics. Therefore, adopting a quantum voting protocol will make the blockchain system immune from underlying quantum computing attacks. In 2017, Thapliyal et al. [38] proposed an efficient quantum binary voting protocol by deploying controlled deterministic secure quantum communication (CDSQC) with a permutation of qubits. By exploiting the Bell state, the operation on a qubit can perform a voting ballot. By adopting this quantum voting protocol in QPoA, voting on the new block can be achieved post-quantum securely.

QPoA adopts a quantum voting scheme based on CDSQC with a permutation of qubits [38] for voting on a candidate block. The candidate block will be accepted and included in the blockchain if it gets more than half of the validating nodes’ affirmative votes. Otherwise, it will be discarded. Specifically, the leader node (for instance, David) publishes the new block on a bulletin board to enable all validating nodes to vote on it. The quantum voting in QPoA consists of three phases: initializing, voting, and counting. The detailed voting process is as follows.

(1) Initializing

There are *N* blocks in the blockchain ledger, and Charlie is the previous leader node that successfully packaged the latest block $B_{N}$ on the blockchain. Charlie acts as the scrutineer in the voting process. David is the current leader node, and he generates a new block $B_{N+1}$ as the candidate block. David initiates voting on $B_{N+1}$ by sending $B_{N+1}$ to all validating nodes, and David is the tallyman. The voters are all validating nodes except Charlie.

**Figure 6:**
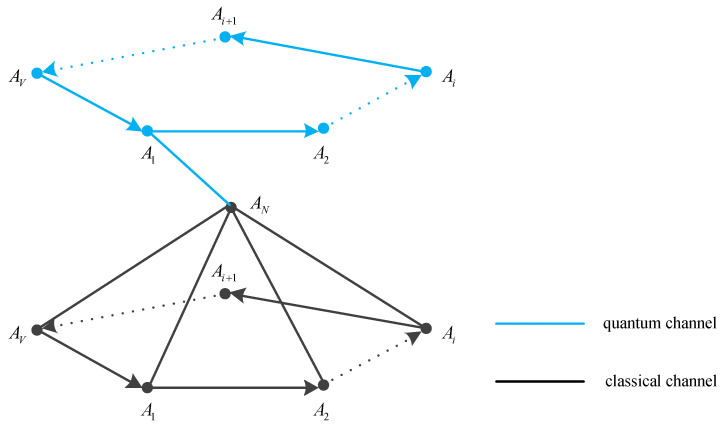
The hybrid network diagram of the multi-party QKD protocol.

**I-Step 1:** The controller, Charlie, sets up a bulletin board to release announcements. The data on the bulletin board cannot be tampered with as all nodes supervise it. By performing a multi-party QKD protocol [30], a secret parameter P is shared among all validating nodes, including David. The sharing of the QHF parameter P is illustrated in Figure 6. David calculates an authentication code $h_{N+1}$ for $B_{N+1}$ by a parameterized QHF [19] with P.$h_{N+1}=QHF(P,B_{N+1})$.Then David broadcasts ($B_{N+1}$,$c_{N+1}$) to all validating nodes, and the integrity of $B_{N+1}$ can be verified by $h_{N+1}$.

**I-Step 2:** Through the QKD protocol [11,25,26,27,28,29,30], David performs identity authentication for all voters. Then David initiates a voting request to all voters by publishing the candidate block $B_{N+1}$ with a deadline that is a time point $t_{end}$. David publishes the voting rule that all voters should obey. That is, performing *I* on the received qubit represents a negative vote, while performing the Pauli gate X represents an affirmative vote.

The Bell states are denoted as follows:

$$\begin{array}{l} |\Phi^{+}\rangle =\frac{1}{\sqrt{2}}\left(|00\rangle +|11\rangle \right)\\ |\Phi^{-}\rangle =\frac{1}{\sqrt{2}}\left(|00\rangle -|11\rangle \right)\\ |\Psi^{+}\rangle =\frac{1}{\sqrt{2}}\left(|01\rangle +|10\rangle \right)\\ |\Psi^{-}\rangle =\frac{1}{\sqrt{2}}\left(|01\rangle -|10\rangle \right) \end{array}$$

Let $I=\left(\begin{array}{cc} 1 & 0\\ 0 & 1 \end{array}\right)$ be the unitary operator, $X=\left(\begin{array}{cc} 0 & 1\\ 1 & 0 \end{array}\right)$ be the X operator.

(2) Voting Phase

**V-Step 1:** Charlie prepares *n* pairs of Bell state particles selected in $\left\{|\Phi^{+}\rangle ,|\Phi^{-}\rangle ,|\Psi^{+}\rangle ,|\Psi^{-}\rangle \right\}$, which is denoted as $\left\{|S_{1}\rangle ,|S_{2}\rangle \right\}$, $|S_{1}\rangle$ represents the sequence of the first qubits and $|S_{2}\rangle$ represents the sequence of the second qubits. Charlie sends the first qubit of the *i*th Bell state to the *i*th voter and prepares an *n*-qubit sequence with the second qubits of all the Bell states, which is denoted as $|S_{2}\rangle$. After that, he performs a permutation operator on each particle in $|S_{2}\rangle$ to generate a new sequence $|{\hat{S}}_{2}\rangle$ and sends $|{\hat{S}}_{2}\rangle$ to David.

**Figure 7:**
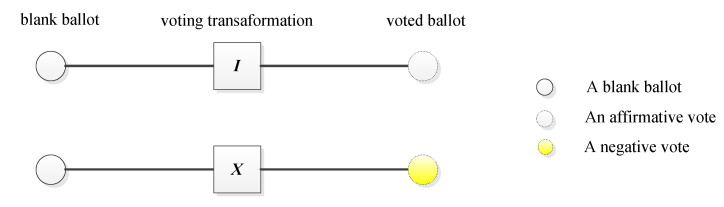
The quantum circuit of quantum voting.

**V-Step 2:** According to the previously established voting rules, every voter casts a vote by performing a corresponding operator on the received qubit. A voter operates *I* on the qubit for a negative vote or operates *X* on the qubit for an affirmative vote. The quantum circuit of quantum voting is illustrated in Figure 7. Then the voter sends the voting result (the transformed qubit) to David, these transformed qubits formed a new sequence $|{\hat{S}}_{1}\rangle$.

**V-Step 3:** After David receives the qubits of Bell states that carry the ballot information from all voters or the time reaches $t_{end}$, David informs Charlie that the collection of votes is accomplished.

(3) Counting Phase

**Figure 8:**
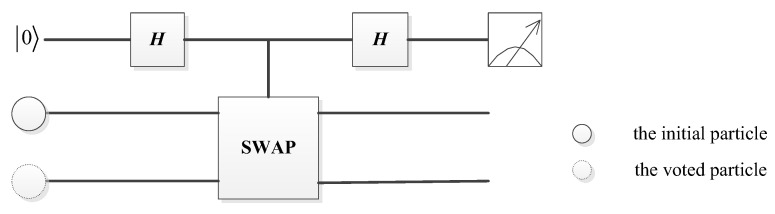
The quantum circuit of the counting phase in quantum voting.

Charlie informs David about the original state and the permutation operator information. Then David figures out that the specific qubit in $|{\hat{S}}_{2}\rangle$ was originally entangled with the qubit in $|{\hat{S}}_{1}\rangle$. After receiving the information from Charlie, David performs Bell measurements on each partner particle to get the voting result cast by every voter, as he already knows the original Bell states. For example, Charlie prepares a Bell state $|\Phi^{+}\rangle =\frac{1}{\sqrt{2}}(|00\rangle +|11\rangle )$ and sends the first particle to a voter. If the voter performs an X operator on the first particle, and Charlie performs an I operator on the second particle. After measurement by David, the outcome is $|\Psi^{+}\rangle =\frac{1}{\sqrt{2}}(|10\rangle +|01\rangle )$, Charlie announces the initial state $|\Phi^{+}\rangle =\frac{1}{\sqrt{2}}(|00\rangle +|11\rangle )$. Based on his measurement result and the initial state, David can deduce that the voter performed operation X, i.e., voted in approval. Due to the statistical nature of quantum measurements, judging whether two quantum states are identical is not as straightforward as comparing two classical numbers. In order to tell whether two quantum states are the same, the Quantum Swap Test Circuit (QSTC) proposed by Buhrman is used, as shown in Figure 8.

Ultimately, by counting all votes, David publishes every voting result on the bulletin board and calculates the number of affirmative votes $V_{a}$. If $V_{a}>\frac{V}{2}$, block $B_{N+1}$ will be accepted and included in the blockchain. After the inclusion of block $B_{N+1}$, the timestamp of the (*N* + 1)-th block $t_{N+1}$ will be used as a seed of QRNG to elect the next leader node from the left validating nodes in LV.

#### 2.2.3. Generation of LV in a Consensus Round

By conventional PoA, generating a new block can be delayed or even fail because of some anomalous or malicious behavior of the leader node. Generally, the leader node will not perform malicious behaviors. However, if the reward brought by malicious behaviors is much greater than the loss of authority, the leader node may harm the blockchain system at the risk of having that authority revoked. To address this issue, QPoA introduces a dynamic updating mechanism for validating nodes based on the credit scores of the nodes.

We define five types of nodes in a blockchain system with QpoA, as reported in Table 2, and we introduce a behavior-scoring mechanism in QpoA. The behaviors of the nodes are quantified by scores. A score will be set for each behavior based on how much the behavior damages the system, and a node will be assigned a corresponding score after performing each behavior.

We define B types of malicious behaviors for the nodes, denoted by $M_{b}(1\leq r\leq B)$. Each $M_{b}$ is assigned a weight $W_{b}$,$\sum_{b=1}^{B}W_{b}=1$. Furthermore, for a specific malicious behavior $M_{b}$, a threshold $T_{b}$ is assigned, which means that the behavior $M_{b}$ can be performed no more than $T_{b}$ times. If a node has a behavior $M_{b}$ for more than $T_{b}$ times, it will be demoted as a malicious node. The malicious behaviors are introduced as follows.

b=1 (fpb), represents the failure of proposing or including a valid block into the blockchain when it is a leader node $W_{b}=w_{1},T_{b}=t_{1}.$

b=2 (fvb), represents the failure of validating a valid block, i.e., giving a veto on a valid block $W_{b}=w_{2},T_{b}=t_{2}.$

b=3 (fvt), represents the failure of submitting a valid transaction, i.e., submitting an invalid transaction to the leader node $W_{b}=w_{3},T_{b}=t_{3}.$

b=4 (otherf), represents the communication response failure or offline status, or other failures $W_{b}=w_{4},T_{b}=t_{4}.$

(1) $$S_{mi}=\sum_{b=1}^{B}\left(\frac{t_{bi}}{T_{b}}\times W_{b}\right),(0\leq t_{bi},1\leq b\leq B,0<W_{b}<1).$$

where $t_{ri}$ denotes the number of times behavior *r* is implemented by node i.

In QpoA, we develop an updating mechanism. Updating the validating nodes includes excluding old validating nodes and including new ones.

After a consensus round completes, all validating nodes have performed the role of leader node, and the LV will be updated. Each validating node will obtain two behavior scores, for node i, $S_{mi}$ denotes the malicious behavior score. For node i, if it has malicious behavior b more than $T_{b}$ times or $S_{mi}>S_{threshhold}$, it will be excluded from the LV and will be demoted as a malicious node, which cannot be elected as a validating node. After checking all validating nodes, the malicious nodes will be excluded, and the existing validating nodes and all authority nodes will form the set of validating node candidates for the next consensus round.

The behavior score is introduced to evaluate the behaviors of a node and is used to dynamically elect validating nodes to participate in each consensus round to enhance the degree of “decentralization”.

**Figure 9:**
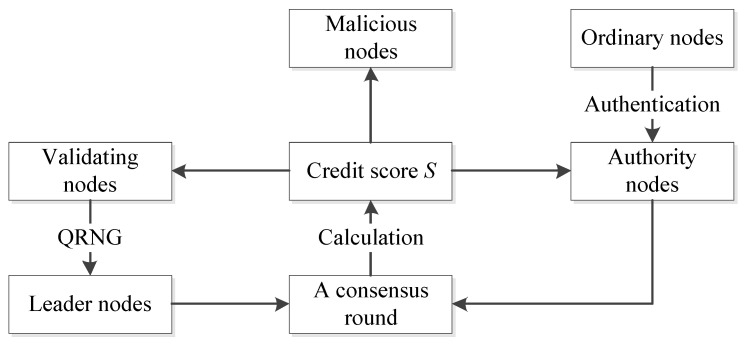
Updating process of the validating nodes in QPoA.

After calculation, malicious nodes will be excluded and new authority nodes will be added to form the LV for the next consensus round. The updating process of the validating nodes in QPoA is presented in Figure 9.

All validating nodes share their secret parameter with each other through a secure quantum channel. The algorithm in Algorithm 1 will output a new reordered list from the LV using their parameters. The nodes in the leader node list will act as leader nodes in turn.

Compared to PoA, QPoA is more reliable since the validating node’s reputation is fully taken into consideration. With the evaluation of malicious behaviors by scores, maliciousness can be reduced and the trustworthiness of the validating node is improved in the consensus.

In summary, QPoA improves on the PoA consensus mechanism in three aspects. First, QPoA discards the classical voting method in PoA and adopts a quantum binary voting protocol [38] instead, securing the voting process from quantum computing attacks. Second, QPoA deploys a quantum multi-party secure computing protocol [37] to generate a shared key P that is used to share the leader node’s identity secretly. The Knuth shuffling method is adopted in the algorithm for the leader node selection, reordering the LV to hide the identity of the leader node from attacks. Third, by auditing the behavior of the validating nodes and quantifying malicious behaviors with a score, the authority of nodes can be dynamically adjusted according to their scores, which further improves the trustworthiness of the validating nodes and the efficiency of consensus.

## 3. The Transaction Process in QSB

In QSB, there are three parties participating in a transaction, the transaction initiator—Alice—the transaction receiver—Bob—and the trusted validating node—Trent. Alice signs the transaction and generates a signature, Trent verifies the signature, and, if verification passes, Bob accepts the transaction. Otherwise, the transaction will be discarded. The security of transaction initiation and verification is governed by the physical properties of quantum signatures. In this section, we will describe the complete process of a transaction in detail, including the four phases of initialization, key generation, signing, and verification. Moreover, with the virtue of QPoA, the trustworthiness of validating nodes is guaranteed, the trusted validating nodes act as verifiers for transactions, ensuring the security of transactions in QSB.

### 3.1. Initialization Phase

Let $H=\frac{1}{\sqrt{2}}\left(\begin{array}{cc} 1 & 1\\ 1 & -1 \end{array}\right)$ be the Hadamard operator. The effect of the H-gate operating on a particle is equivalent to multiplying its corresponding matrix by the particle’s quantum state vector. The quantum logic gate H operation is depicted as follows:

$$H|0\rangle =\frac{1}{\sqrt{2}}\left(|0\rangle +|1\rangle \right)=|+\rangle ,\;H|1\rangle =\frac{1}{\sqrt{2}}\left(|0\rangle -|1\rangle \right)=|-\rangle ,\;H^{2}=I.$$

where I is the unit operator. For any strings $x=\left(x_{1},x_{2},\ldots ,x_{n}\right)$ and $y=\left(y_{1},y_{2},\ldots ,y_{n}\right)$, we define that $x⊕y=\left(x_{1}⊕y_{1},x_{2}⊕y_{2},\ldots ,x_{n}⊕y_{n}\right)$, where “⊕” denotes the addition operation under modular two.

In this phase, Alice and Bob secretly saved a one-way function $G:{\left\{0,1\right\}}^{*}\to {\left\{0,1\right\}}^{n}$ as their master key, and the output of G is uniformly distributed. Suppose that the identification of Alice is $ID_{A}\in {\left(0,1\right)}^{n}$, the identification of Bob is $ID_{B}\in {\left(0,1\right)}^{n}$, and transaction information Tx is encoded into a binary string $m=\{m_{i}\}\in {\left(0,1\right)}^{n},i=1,2,\ldots ,n$.

### 3.2. Key Generation Phase

Alice, Bob, and Trent share their private keys by performing the following steps.

**K-step 1:** Alice calculates $p=G\left(ID_{A}\right)$ with the master key G. In the same way, Bob calculates $q=G\left(ID_{B}\right)$.

**K-step 2:** By performing the QKD protocol [11,25,26,27,28,29,30], a temporary random secret key $z=\left(z_{1},z_{2},\ldots ,z_{n}\right)$ is shared between Alice and Bob. Bob calculates $l_{1}⊕z={l^{\prime }}_{1}$, $l_{2}⊕z={l^{\prime }}_{2}$, and Bob publicly announces ${l^{\prime }}_{1}$ and ${l^{\prime }}_{2}$. Based on the secret pad z, Alice decrypts the OTP ciphertexts ${l^{\prime }}_{1}$ and ${l^{\prime }}_{2}$, then gets their private parameters $l_{1}=z⊕{l^{\prime }}_{1}$ and $l_{2}=z⊕{l^{\prime }}_{2}$.

**Figure 10:**
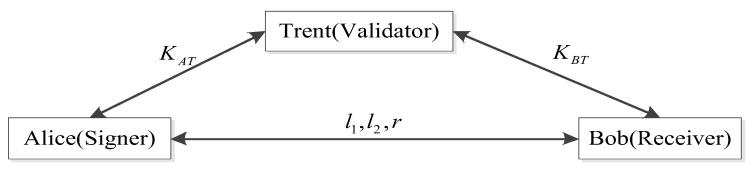
Structure of QS.

**K-step 3:** By performing the QKD protocol [11,25,26,27,28,29,30], the private key $K_{AT}$ is shared between Alice and Trent, and the private key $K_{BT}$ is shared between Bob and Trent. The structure of QS is shown in Figure 10.

### 3.3. Signing Phase

**S-step 1:** Alice secretly generates an n-bits parameter $r=\left(r_{1},r_{2},\ldots ,r_{n}\right)$, and Alice computes

(2) $$f=m⊕ID_{A}⊕r⊕K_{AT}⊕l_{1}.$$

(3) $$|\alpha \rangle =H^{p}|f\rangle .$$

(4) $$u=r⊕l_{1}⊕m.$$

(5) $$|\delta \rangle =H^{K_{AT}}|u\rangle .$$

The quantum signature on m is $|\delta \rangle :={⊗}_{i=1}^{n}|\delta_{i}\rangle$.

**S-step 2:** Alice generates l(l>>2n) decoy particles, which are randomly distributed in {$|0\rangle ,|1\rangle ,|+\rangle ,|-\rangle$} to check for eavesdropping. These decoy particles will be randomly inserted into the sequence $|\alpha \rangle$ and $|\delta \rangle$, then the corresponding particle sequence $|\alpha^{\prime }\rangle$ and $|\delta^{\prime }\rangle$ are generated. After that, $|\alpha^{\prime }\rangle$ is sent to Bob and $|\delta^{\prime }\rangle$ is sent to Trent.

**S-step 3:** Bob secretly generates an n-bits parameter $s=\left(s_{1},s_{2},\ldots ,s_{n}\right)$, and Bob computes

(6) $$g=m⊕ID_{B}⊕s⊕K_{BT}⊕l_{2}.$$

(7) $$|\beta \rangle =H^{q}|g\rangle .$$

(8) $$v=s⊕l_{2}⊕m.$$

(9) $$|\gamma \rangle =H^{K_{BT}}|v\rangle .$$

**S-step 4:** Bob generates l(l>>2n) decoy particles randomly distributed in {$|0\rangle ,|1\rangle ,|+\rangle ,|-\rangle$} to check for eavesdropping. These decoy particles are randomly inserted into the sequence $|\beta \rangle$ and $|\gamma \rangle$, then the corresponding particle sequence $|\beta^{\prime }\rangle$ and $|\gamma^{\prime }\rangle$ are generated. After that, $|\beta^{\prime }\rangle$ is sent to Alice and $|\gamma^{\prime }\rangle$ is sent to Trent.

**S-step 5:** After confirming that $|\alpha^{\prime }\rangle$ has been received by Bob, $|\beta^{\prime }\rangle$ has been received by Alice, and $|\delta^{\prime }\rangle$ and $|\gamma^{\prime }\rangle$ have been received by Trent, the initial states and location information of the decoy particles in $|\alpha^{\prime }\rangle$ and $|\delta^{\prime }\rangle$ will be announced by Alice. Moreover, the initial states and location information of the decoy particles in $|\beta^{\prime }\rangle$ and $|\gamma^{\prime }\rangle$ will be announced by Bob. After that, Alice and Bob measure the decoy particles in the provided positions with the corresponding basis and compare the initial states with the measured results, respectively. If there is no error in checking, Bob proceeds to the next step. Otherwise, the protocol will be restarted.

### 3.4. Verification Phase

**V-step 1:** After Alice has recovered $|\beta \rangle$, she calculates $q=G\left(ID_{B}\right)$, and according to q, she measures $|\beta \rangle$. If q=0, she will measure $|\beta \rangle$ with {$|0\rangle ,|1\rangle$}, and if q=1, she will measure $|\beta \rangle$ with {$|+\rangle ,|-\rangle$}. Additionally, if $|\beta \rangle =|0\rangle$ or $|+\rangle$, then Alice gets g=0, and if $|\beta \rangle =|1\rangle$ or $|-\rangle$, then Alice gets g=1.

Similarly, after Bob recovers $|\alpha \rangle$, he calculates $p=G\left(ID_{A}\right)$, and according to p, he measures $|\alpha \rangle$. If p=0, he will measure $|\alpha \rangle$ with {$|0\rangle ,|1\rangle$}, and if p=1, he will measure $|\alpha \rangle$ with {$|+\rangle ,|-\rangle$}. Moreover, if $|\alpha \rangle =|0\rangle$ or $|+\rangle$, then Bob gets f=0, and if $|\alpha \rangle =|1\rangle$ or $|-\rangle$, then Bob gets f=1.

**V-step 2:** Alice calculates

(10) $$s^{\prime }=g⊕m⊕ID_{B}⊕K_{BT}⊕l_{2}.$$

According to Equation (10), she obtains

(11) $$c=s^{\prime }⊕K_{BT}⊕l_{2}.$$

Then she sends c to Trent.

**V-step 3:** Bob calculates

(12) $$r^{\prime }=f⊕m⊕ID_{A}⊕K_{AT}⊕l_{1}.$$

According to Equation (12), he gets

(13) $$d=r^{\prime }⊕K_{AT}⊕l_{1}.$$

Then he sends d to Trent.

**V-step 4:** Trent measures $|\delta \rangle$, according to $K_{AT}$. If $K_{AT}=0$, he will measure $|\delta \rangle$ with {$|0\rangle ,|1\rangle$}, and if $K_{AT}=1$, he will measure $|\delta \rangle$ with {$|+\rangle ,|-\rangle$}. In addition, if $|\delta \rangle =|0\rangle$ or $|+\rangle$, then Trent sets $u^{\prime }=0$, and if $|\delta \rangle =|1\rangle$ or $|-\rangle$, then Trent sets $u^{\prime }=1$.

Similarly, Trent measures $|\gamma \rangle$, according to $K_{BT}$. If $K_{BT}=0$, he will measure $|\gamma \rangle$ with {$|0\rangle ,|1\rangle$}, and if $K_{BT}=1$, he will measure $|\gamma \rangle$ with {$|+\rangle ,|-\rangle$}. If $|\gamma \rangle =|0\rangle$ or $|+\rangle$, then Trent sets $v^{\prime }=0$, and if $|\gamma \rangle =|1\rangle$ or $|-\rangle$, then Trent sets $v^{\prime }=1$. Trent secretly keeps $u^{\prime }$ and $v^{\prime }$.

**V-step 5:** According to sharing key $K_{AT}$ and $K_{BT}$, Trent computes

(14) $$c⊕d=\mu_{1}.$$

(15) $$u^{\prime }⊕v^{\prime }⊕K_{AT}⊕K_{BT}=\mu_{2}.$$

**Figure 11:**
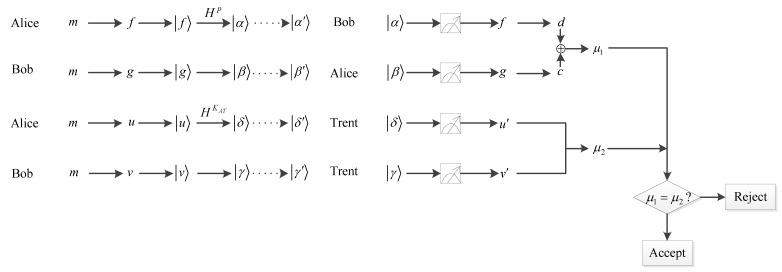
Process of IQS.

Finally, Trent compares the outcomes of Equations (14) and (15), and if $\mu_{1}=\mu_{2}$, the signature is valid. Trent publishes $\mu_{1}$ and $u^{\prime }⊕v^{\prime }$, and Alice and Bob publish *s*, *r*, *l*_1_, *l*_2_. Otherwise, it will be abandoned. The whole process of signing and verification for a transaction is shown in Figure 11.

Alice initiates a transaction *Tx* with Bob, Trent is a validating node and acts as the designated verifier to verify *Tx* by IQS. If the verification succeeds, *Tx* will be sent to the leader node David by Trent. David collects verified transactions from all validating nodes, adds them to the transaction queue, sorts them by their timestamp, and validates them one by one. A valid transaction will be packaged into a candidate block while an invalid one will be rejected. After valid transactions accumulate to a certain number or time reaches $t_{end}$, the candidate block $B_{N+1}$ will be published to all validating nodes for voting, if $B_{N+1}$ is accepted by the voting result, then *Tx* is included in the chain.

## 4. Security and Analysis of QSB

QSB can effectively resist common attacks against blockchain systems, such as 51% attacks, double spending attacks, and Sybil attacks.

Regarding 51% attacks, in QPoA, an attacker is required to control over 51% of validating nodes, which is quite different from the case in the PoW-based blockchain where an attacker is required to obtain 51% of the computational power of the whole network. It is almost impossible for an attacker to obtain control of such a large percentage of validating nodes in a permissioned blockchain network, which is more difficult than obtaining computational power. For example, in a PoW-based network, an attacker can increase the computation power of its controlled nodes to improve the chance of obtaining bookkeeping rights, while this makes no sense for QPoA because QPoA is computation independent, the computational power of the node has no effect on the block generation decisions.

Double spending attacks are eliminated in QSB by the leader node, as all verified transactions will be validated by the leader node one by one in a strict chronological order, any spent assets will be taken into consideration when validating a transaction. Additionally, even if an invalid double-spending transaction has been packaged in a candidate block, before the generation of this block, during the voting process participated in by all validating nodes, the invalid transaction will easily be found and the block will be rejected by voting.

As for Sybil attacks, QSB conceals the identity of the leader node by the leader node selection algorithm, the timestamp of a block is unpredictable and the randomness of QRNG also guarantees the randomization of the leader node. Furthermore, the identity of the leader node is shared among all validating nodes through a secure quantum channel with the help of public key P generated by secure multi-party QKD protocol [37].

### 4.1. Security of QPoA Consensus Mechanism

The QPoA can initially set up authorized nodes called validating nodes with transaction verification and voting rights. A new transaction needs to be verified by the validating nodes before its publishment on the chain. For a new validating node enrolled at a later stage, the current validating nodes can also decide whether to allow it to join the LV by voting. The QPoA can greatly increase the speed because it reduces the computational cost of reaching consensus.

In addition, due to the adoption of a quantum voting protocol that is immune from quantum computing attacks, QPoA is post-quantum secure.

### 4.2. Security of Transactions

In QSB, the security of a transaction is guaranteed by IQS. This section conducts a detailed security analysis of IQS, which includes unforgeability and resistance to repudiations and impersonation attacks.

#### 4.2.1. Information-Theoretic Security of IQS

The quantum ciphertext should be information-theoretically indistinguishable for a secure quantum cryptosystem under a quantum chosen-plaintext attack (IND-CPA) [39].

**Theorem 1**  [40]**.** *For all plaintexts* x *and* y*, let the density operators of the cipher states* E(x) *and* E(y) *be* $\rho_{x}$ *and* $\rho_{y}$*, respectively. A quantum public-key encryption scheme is said to be information-theoretically indistinguishable if, for every positive polynomial p(* *·) and every sufficiently large* n*,*

(16) $$D\left(\rho_{x},\rho_{y}\right)<1/p\left(n\right).$$

Thus, based on Theorem 1, our scheme has ciphertext indistinguishability under quantum IND-CPA.

**Proof.**  Let $|\alpha^{*}\rangle$ and $|\alpha \rangle$ be the ciphertexts of different plaintexts m* and m, respectively. For an adversary Eve, the density operators of $|\alpha^{*}\rangle$ and $|\alpha \rangle$ should take the possible values of private keys r. Note that f satisfies Equation (2), in which r, $K_{AT}$, $l_{1}$, and m have uniform distributions. Hence, the density operator of $|\alpha \rangle$ >can be computed as follows

(17) $$\begin{array}{l} \rho_{r,m}=\frac{1}{2^{4n}}{⊗}_{i=1}^{n}\sum_{r,l_{1},K_{AT}}|\alpha \rangle \langle \alpha |\\ =\frac{1}{2^{4n}}\sum_{a,P_{A},E}{⊗}_{i=1}^{n}\left(H^{P}|f\rangle \langle f|H^{P}\right)\\ =I/2^{n} \end{array}$$

Similarly, the density operator of $|\alpha^{*}\rangle$ can be computed as follows

(18) $$\begin{array}{l} \rho_{r^{*},m^{*}}=\frac{1}{2^{4n}}{⊗}_{i=1}^{n}\sum_{r,l_{1},K_{AT}}|\alpha^{*}\rangle \langle \alpha^{*}|\\ =\frac{1}{2^{4n}}\sum_{a,P_{A},E}{⊗}_{i=1}^{n}\left(H^{p}|f^{*}\rangle \langle f^{*}|H^{p}\right)\\ =I/2^{n} \end{array}$$

Let $|b^{*}\rangle$ and $|b\rangle$ be the ciphertexts of different plaintexts m* and m, respectively. For an adversary Eve, the density operators of $|b^{*}\rangle$ and $|b\rangle$ should take the possible values of private keys s. Note that s satisfies Equation (6), in which s, $K_{BT}$, $l_{2}$, and m have uniform distributions. Hence, the density operator of $|b\rangle$ can be computed as follows

(19) $$\begin{array}{l} \rho_{s,m}=\frac{1}{2^{4n}}{⊗}_{i=1}^{n}\sum_{s,l_{2},K_{BT}}|\beta \rangle \langle \beta |\\ =\frac{1}{2^{4n}}\sum_{s,l_{2},K_{BT}}{⊗}_{i=1}^{n}\left(H^{q}|g\rangle \langle g|H^{q}\right)\\ =I/2^{n} \end{array}$$

Similarly, the density operator of $|\beta^{*}\rangle$ can be computed as follows

(20) $$\begin{array}{l} \rho_{s^{*},m^{*}}=\frac{1}{2^{4n}}{⊗}_{i=1}^{n}\sum_{s,l_{2},K_{BT}}|\beta^{*}\rangle \langle \beta^{*}|\\ =\frac{1}{2^{4n}}\sum_{s,l_{2},K_{BT}}{⊗}_{i=1}^{n}\left(H^{q}|g^{*}\rangle \langle g^{*}|H^{q}\right)\\ =I/2^{n} \end{array}$$

Similarly, as Equations (17)–(20), it follows that

(21) $$D\left(\rho_{r,m},\rho_{r^{*},m^{*}}\right)=0,$$

(22) $$D\left(\rho_{s,m},\rho_{s^{*},m^{*}}\right)=0.$$

According to Theorem 1, these bounds make IQS theoretically information IND-CPA secure. □

#### 4.2.2. Secrecy of the Private Key

In IQS, Trent is a trusted node who will never disclose the private key of Alice and Bob. Hence, the adversary, Eve, tries to retrieve the private key only from the public information.

First, Eve cannot retrieve the private key from the identity. In the key generation phase, Alice and Bob generate their private keys $p=G\left(ID_{A}\right)$, $q=G\left(ID_{B}\right)$ with the master key G. As G is a one-way function, if G is chosen as a random one-way function, Eve can succeed in guessing G with a negligible probability $\frac{1}{(2n)!}$. Therefore, it is infeasible for Eve to retrieve the private key from the identity.

Second, in the key generation phase, it is infeasible for Eve to retrieve private parameters from ${l^{\prime }}_{1}$ and ${l^{\prime }}_{2}$ announced by Alice and Bob. Note that $l_{1}⊕z={l^{\prime }}_{1}$, $l_{2}⊕z={l^{\prime }}_{2}$ where the secret z is generated by performing the secure QKD protocol. Therefore, Eve cannot get z due to the unconditional security of the QKD protocol. Here,z acts as a random pad, ${l^{\prime }}_{1}$ and ${l^{\prime }}_{2}$ can be seen as OTP ciphertexts [18] of the private parameters $l_{1}$ and $l_{2}$. Since it is guaranteed by the unconditional security of OTP [24], it is unworkable for Eve to retrieve the private key $l_{1}$ and $l_{2}$ from the OTP ciphertext ${l^{\prime }}_{1}$ and ${l^{\prime }}_{2}$ without knowing the secret pad z. Therefore, the security of secret keys such as $K_{AT}$, $K_{BT}$, s, r, $l_{1}$, $l_{2}$ is provided by the unconditional information theoretical security of QKD protocols and OTP.

Finally, Eve cannot get the quantum ciphertexts $|\alpha \rangle$ and $|\beta \rangle$, which contain the information of the signer’s private key. In IQS, as decoy particles are utilized in eavesdropping checking in the quantum channel, Eve’s eavesdropping attacks will necessarily cause disturbances to the decoy particles, which will be found by the receiver easily in the signing and signature verification phases. Even if Eve obtains the quantum ciphertexts $|\alpha \rangle$ and $|\beta \rangle$ by chance, it is unworkable for Eve to retrieve the private key $K_{AT}$ and $K_{BT}$ from $|\alpha \rangle$ and $|\beta \rangle$ which is guaranteed by the information-theoretical IND-CPA security of IQS. Therefore, it is unworkable for Eve to retrieve the signer’s private key.

#### 4.2.3. Security against Forgery

The unforgeability of QSB is analyzed in this section. According to the signing phase, the private key $K_{AT}$ and the secret pad $l_{1}$ are necessary for signing a message. However, according to the security analysis of the private key presented in Section 4.2.2, Eve cannot retrieve the private key $K_{AT}$ and the secret pad $l_{1}$ from the public information. Similarly, according to S-step3 in Section 3.1, the private key $K_{BT}$ and the secret pad $l_{2}$ are necessary for signing a message. However, according to the security analysis of the private key in Section 4.2.2, Eve cannot retrieve the private key $K_{BT}$ and the secret pad $l_{2}$ from the public information. Therefore, it is infeasible for Eve to forge a valid signature without $K_{AT}$, $K_{BT}$ and secret pads $l_{1}$ and $l_{2}$. Even Trent cannot forge the signer’s signature since he knows nothing about the secret pads $l_{1}$ and $l_{2}$, which are only possessed by Alice and Bob. Similarly, the receiver, Bob, cannot forge the signer’s signature since he knows nothing about the key $K_{AT}$ and private parameter r that Alice secretly generates.

#### 4.2.4. Security against Repudiations

Non-repudiation means that in a valid transaction, the signer, Alice, cannot deny the fact that she has signed the transaction, and the receiver, Bob, cannot deny the fact that he has accepted the verified signature.

In a typical transaction, the validating node Trent is a trusted third party that neither reveals the signer’s private key nor impersonates the signer to sign any message. From Section 4.2.3, we know that the proposed QS is secure against forgery. Therefore, as long as the verification succeeds, the validity of the signature should not be denied by either the signer or the receiver.

In general, for a verified transaction, the state of the QS is changed after verification. This means both the signer and the receiver will lose the QS. Specifically, in our scheme, once the QS passes the verification by Trent, both Alice and Bob will lose the QS $|\delta \rangle$. So, the signer Alice may deny the fact that she has ever generated a QS, while the receiver Bob may deny the fact that he has ever received a QS. We call this a disputation of a lost quantum signature. Most of the existing QS schemes cannot arbitrate this kind of disputation, while the proposed IQS can arbitrate this kind of disputation.

Noting that the signature proof (m, $ID_{A}$, $ID_{B}$, s, r, $l_{1}$, $l_{2}$, $\mu_{1}$, $u^{\prime }⊕v^{\prime }$) is stored by Trent. Once the signer Alice denies the fact that she has ever generated the QS on m, Bob can submit a re-verification request to Trent with partial transaction data (m, $ID_{A}$, $ID_{B}$, s, $l_{1}$, $l_{2}$). In response to the request, Trent recovers the corresponding signature proof (m, $ID_{A}$, $ID_{B}$, s, r, $l_{1}$, $l_{2}$, $\mu_{1}$, $u^{\prime }⊕v^{\prime }$) in which *r* is published by Alice, *s* is published by Bob, $l_{1}$, $l_{2}$ are co-published by Alice and Bob during the verification phase. Then Trent recovers the private key $K_{AT}$, $K_{BT}$ from $ID_{A}$, $ID_{B}$ respectively. Next, by Equations (2) and (6), Trent calculates *f* and *g* respectively. Moreover, according to Equations (10)–(14) and Equation (15), Trent performs the following two calculations respectively.

(23) $$\mu_{1}*=c⊕d=g⊕ID_{B}⊕f⊕ID_{A},$$

(24) $$\mu_{2}*=u^{\prime }⊕v^{\prime }⊕K_{AT}⊕K_{BT}.$$

After that, Trent compares $\mu_{1}*$, $\mu_{2}*$ with $\mu_{1}$. If $\mu_{1}*=\mu_{2}*=\mu_{1}$, Trent can confirm that Alice has generated the QS for Bob since only Alice can work out the valid f with the private key $K_{AT}$ and r. Similarly, Bob cannot deny the fact that he has ever received the valid QS because Trent has stored the signature proof (m, $ID_{A}$, $ID_{B}$, s, r, $l_{1}$, $l_{2}$, $\mu_{1}$, $u^{\prime }⊕v^{\prime }$). According to the partial transaction data (m, $ID_{A}$, $ID_{B}$, r, $l_{1}$, $l_{2}$) submitted by Alice and the signature proof (m, $ID_{A}$, $ID_{B}$, s, r, $l_{1}$, $l_{2}$, $\mu_{1}$, $u^{\prime }⊕v^{\prime }$), Trent can calculate $\mu_{1}*$, $\mu_{2}*$ and verify whether $\mu_{1}*=\mu_{2}*=\mu_{1}$ as above. If $\mu_{1}*=\mu_{2}*=\mu_{1}$, Bob cannot deny the fact that he has ever received the valid QS, since only Bob can work out the valid g with the private key $K_{BT}$ and s. In summary, our scheme not only realizes transaction data non-repudiation but is also able to arbitrate the potential disputation of lost quantum signatures, which cannot be achieved in most QS schemes.

### 4.3. Security of Transaction Records

A post-quantum secured QHF is utilized in the block structure to maintain the data integrity of transaction records. As the multi-party QKD protocol provides secure sharing of QHF parameters among all the validating nodes. Without the parameter *p*, an adversary can not implement the preimage collision attack for the QHF. Due to the adoption of confidential quantum walk parameters, QHF is resistant to preimage-collision attacks faced by classical hash functions. In this way, transaction records on the blockchain ledger can be protected from tampering.

Overall, the post-quantum security of QSB is enhanced from three aspects: consensus mechanism QPoA, transaction digital signature IQS, and tamper-proof QHF.

### 4.4. Analysis of the Efficiency of QSB

First, it is well known that quantum resources are expensive at the current state of the art and an efficient transaction should use as few quantum resources as possible. In the literature [4], in order to complete the verification of a single transaction, the distribution of O(*n*^2^) copies of quantum public key among *n* nodes is implemented by O(*n*^2^) times communications, which consumes large quantum resources and quantum communication resources. The high overheads of quantum resources in both storage and communication will lead to a reduction in transaction efficiency. While in QSB, a transaction requires only the distribution of O(1) copies of quantum keys among three nodes and it is implemented by O(1) times communications, which largely saves quantum storage resources and quantum communication resources.

Second, the QPoA maintains the LV for new block generation by a rigorous and standardized vetting process, which is fair and transparent. The generation of a new LV is through a dynamic updating mechanism that can maintain the blockchain performing well during a consensus round, the time complexity of a consensus round in QSB is O(*V*), while that of QDPoS is O(n) with *n* > *V*. QSB is also Byzantine fault tolerant, the tolerance rates of the faulty nodes is *n*/2, which is a comparable performance to the literature [4].

Third, in other quantum blockchain schemes, the nodes separately generate new blocks on their own, which is not robust. While in QSB, as the block is totally classical information that can be broadcast in a classical channel easily and no extra quantum information is required to be sent point-to-point, accelerating the block generation process. While Refs. [12,41] send quantum data through quantum channels for verification with an extra consumption of 2*n* qubits and n qubits, respectively.

In addition, in the QDPoS scheme, the election of one-time representative nodes is implemented through quantum voting with time complexity O(*n*). While in QSB, the election of validating nodes is accomplished by QPoA without any quantum resource consumption, the consensus time complexity of QSB is just O(1). From the perspective of quantum voting, our scheme is more efficient than Ref. [4].

### 4.5. Comparison

In QDPoS, the elected representative nodes take turns generating new blocks, this exposes the identity of the bookkeeping node which may attract attacks. Different from QDPoS, QPoA elects the leader node from the LV through a randomized algorithm with QRNG, which fully hides the identity of the leader node and protects the blockchain from centralized attacks, which may lead to a single point of failure.

As to the storage structure of blocks, [4] stores blocks in quantum states, which is expensive and not convenient for data validation as a quantum-state block can be used only once and collapse. While in QSB, blocks are stored in classical states, the storage costs are low and data can be reused for replication and validation, enabling the blockchain system to be more practical. The comparisons of quantum blockchain schemes are made in Table 3, demonstrating that QSB achieves a better balance of post-quantum security, practicality, and efficiency.

## 5. Application Scenario of QSB

**Figure 12:**
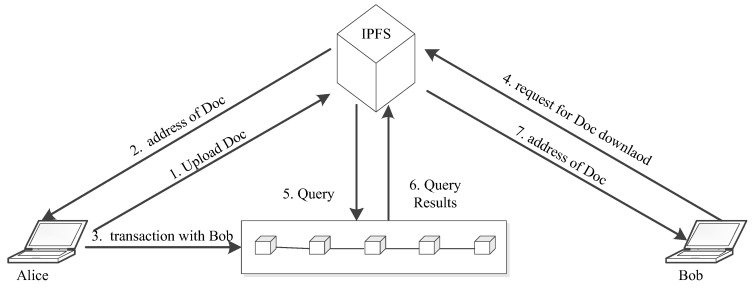
Digital document exchange based on QSB.

The digital document exchange application scenario based on QSB is shown in Figure 12. A document is denoted as Doc, Alice is a data owner, and Bob is a data user. After Alice uploads Doc to the interplanetary file system (IPFS) storage system, she will receive the address of Doc from the storage system. Then Alice and Bob submit a transaction to the QSB. The transaction record includes the following information: sender (Alice), receiver (Bob), verifier, Doc, and timestamp. After the transaction has been recorded on the QSB, Bob is authorized to download the document Doc while an illegal user is forbidden to do so. After that, when Bob sends a download request to the storage platform, the storage platform will query the QSB for relevant transactions. If Bob is a receiver of Doc, the address of Doc will be sent to Bob. Then Bob can succeed in downloading Doc by uploading the required address. Otherwise, Bob’s request will be rejected.

## 6. Conclusions

This paper proposes an IQS scheme, which can leave proof after verification, where the other nodes validate the signature after verification by proof. Second, we improve the conventional PoA from three aspects, i.e., we define five types of nodes with different permissions in the blockchain system, set an updating mechanism for validating nodes based on behavior scoring, and introduce QRNG to conceal the identity of leader nodes. Third, a QSB scheme is proposed using the IQS and the QPoA. Extensive security analysis demonstrates that our method affords an advantage in security against quantum computing attacks compared to conventional blockchains. Finally, we discuss the digital document exchange application scenario based on QSB.

In conclusion, the developed QSB scheme presents a feasible solution for quantum-secured blockchain systems through a quantum strategy. Although it is somewhat expensive to implement, we believe the overhead will decrease with the advancement of quantum technologies. Overall, our work will enrich the research of quantum blockchain in the quantum era.

## Figures and Tables

**Table 1:** Notation table.

Symbol	Definition
*Tx*	A transaction
*m*	A binary string
*N*	Number of blocks in current blockchain ledger
*B_N_*	The *N*th block
*t_N_*	The timestamp of *B_N_*
*M*	The order number of present consensus round
*P_M_*	The public parameter secretly shared by all validating nodes of the *M*th consensus round
LV	The list of validating nodes
*V*	Number of validating nodes in LV
*l*	Number of validating nodes that have already acted as the leader node in a consensus round
$V_{a}$	Number of affirmative votes for a new block
$\left\lfloor x\right\rfloor$	The integer part of *x*
$l_{1}$	A private parameter shared by Alice and Bob
$l_{2}$	A private parameter shared by Alice and Bob
$l_{1}{}^{\prime }$	OTP ciphertext of $l_{1}$
$l_{2}{}^{\prime }$	OTP ciphertext of $l_{2}$
*r*	an n-bits parameter shared by Alice and Bob
$K_{AT}$	the private key shared between Alice and Trent
$K_{BT}$	the private key shared between Bob and Trent

**Table 2:** Permissions of nodes in QpoA.

Nodes	Permissions			
Type of Node (Authority Level)	Propose a New Block	Vote on a New Block	Validate a Transaction	Publish a Transaction
Leader node	√	√	√	√
Validating node	×	√	√	√
Authority node	×	×	√	√
Ordinary node	×	×	×	√
Malicious node	×	×	×	×

**Table 3:** Comparisons of quantum blockchain schemes.

	[10]	[12]	[13]	[14]	[15]	[4]	[16]	[18]	Ours
Drawback	BFT	Entanglement in time	Entanglement in time	quantum swap test circuit has error rate	Lack of a consensus mechanism	storing quantum states on blocks; inefficiency of the QS	storing quantum state public keys on blocks	Nonsecure voting	
Practicality of consensus mechanism	×	-	√	-	-	√	√	√	√
Post-quantum security of voting in consensus mechanism	-	-	-	-	-	√	×	×	√
Post-quantum security of consensus mechanism	√	-	-	-	-	√	×	×	√
Reverification of the digital signature	-	-	×	×	√	×	×	×	√
Not storing quantum states on blocks	√	×	×	√	√	×	×	√	√
Not use quantum swap test circuits	√	√	√	×	√	×	×	√	√

## Data Availability

Not applicable.
